# Revisiting history. The memory of Prof. Hermann J. Muller (1890–1967) in Moscow revived by Helen Muller

**DOI:** 10.3897/CompCytogen.v13i2.37416

**Published:** 2019-07-03

**Authors:** Nina Bulatova

**Affiliations:** 1 A.N. Severtsov Institute of Ecology and Evolution, Russian Academy of Sciences, Moscow 119071, Russia A.N. Severtsov Institute of Ecology and Evolution, Russian Academy of Sciences Moscow Russia

**Keywords:** Early cytogenetics, history of genetics, member of the Russian Academy of Sciences

## Introduction

Last autumn, Moscow geneticists had the pleasure to meet Helen Muller, daughter of Professor Hermann Joseph Muller, American geneticist, educator, and Nobel laureate, best known for his work on the physiological and genetic effects of radiation (X-ray mutagenesis). In search of materials for a book that she is writing about her father, Helen Muller visited the institutions of Russian Academy of Sciences, associated with the presence of H.J. Muller in Moscow in the 1930s.

The scientific career of Hermann J. Muller (1890–1967) began in Columbia University, New York under the supervision of one of the founders of cytogenetics, E.B. Wilson. Muller remained as a postgraduate in the same university through 1912–1916, and in 1918–1920 became an assistant of T.H. Morgan, whose theory of chromosomal heredity (Morgan et al. 1915) they explored together with A.H. Sturtevant and C.B. Bridges. During 1921–1931 Muller was based in University of Texas, where he became professor in 1925. Significant for Muller’s future was his acquaintance with the Russian scientist Nikolai I. Vavilov who came to USA in 1921 as a rising leader of the new Soviet genetics and organizer of the world collection of cultured plants organised according to his newly proposed scheme of homologous genetic series (Vavilov 1920). In 1922, H.J. Muller visited for the first time the institutions in Russia under Vavilov’s influence. Muller brought with him his collections of fruit-fly mutants, introducing genetic studies on *Drosophila* Fallén, 1823 to Russia. In February 1933, H.J. Muller was elected foreign corresponding member of the USSR Academy of Sciences, succeeding the honorary membership of T.H. Morgan in 1932 (www.ras.ru), also at the instigation of N.I. Vavilov.

For four fruitful years Hermann J. Muller worked at the Institute of Genetics, founded by N.I. Vavilov in Leningrad, now St. Petersburg (restoring the traditional name for the city), and moved with his Laboratory of Genes and Mutagenesis to Moscow in 1934. His colleagues were M.L. Bel’govsky, A.A. Prokofieva-Bel’govskaya, Y.J. Kerkis, N.N. Medvedev, K.V. Kosikov and others, well-known Soviet geneticists. Together with his scientific successes, during the last two years of his work in the USSR, Muller devoted much effort to the public defence of the chromosome theory of heredity from the anti-genetic attacks of T. Lysenko. As a consequence of this opposition to Lysenko, H.J. Muller was forced to leave USSR in 1937.

Two places related to Hermann J. Muller’s work in Moscow were visited by his daughter last Autumn. Muller’s lab was situated in a building, which continues to be part of the Russian Academy of Sciences. The old building at number 33 Leninsky Avenue is that one from which academician Nikolai I. Vavilov departed for his last expedition in 1940 and never returned. In protest against the expulsion of N.I. Vavilov, Hermann Muller, already a Nobel laureate (1946), refused membership of the USSR Academy of Sciences. Muller’s name was, however, added to the Academy (www.ras.ru) in recent times (1990). In the 1960s, the rehabilitation of the name of N.I. Vavilov should be proceeded in different ways, including the naming of the new academic Institute of General Genetics, situated close to where Vavilov used to work (www.vigg.ru).

**Figure 1.**
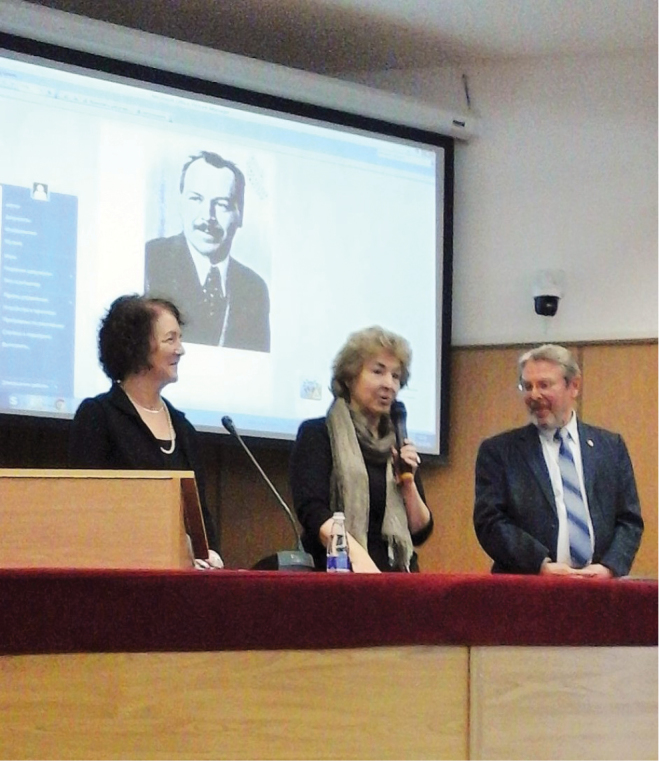
Prof. Helen Muller (left) in the conference hall situated close to the laboratory of her famous father in Moscow. With Dr. Tatjana B. Avrutskaya (N.I. Vavilov Institute) and Academician V.V. Rozhnov, Director of A.N. Severtsov Institute. Photo by N. Bulatova.

Helen Muller, emeritus Professor of Sociology from the University of New Mexico, visited the N.I. Vavilov Institute of General Genetics and memorial museum with portraits of Vavilov’s collaborators, including, of course, H.J. Muller (http://vigg.ru/istorija-instituta/muzei-ni-vavilova/). On 27 September 2018 Helen Muller gave a lecture at the A.N. Severtsov Institute of Ecology and Evolution, at Leninsky Avenue, 33 (http://sev-in.ru/ru/node/804) in the same conference hall familiar to both Vavilov and Muller (Fig. 1).
